# Implementation science in cancer prevention and control: a decade of grant funding by the National Cancer Institute and future directions

**DOI:** 10.1186/s13012-014-0200-2

**Published:** 2015-01-08

**Authors:** Gila Neta, Michael A Sanchez, David A Chambers, Siobhan M Phillips, Bryan Leyva, Laurie Cynkin, Margaret M Farrell, Suzanne Heurtin-Roberts, Cynthia Vinson

**Affiliations:** Division of Cancer Control and Population Sciences, National Cancer Institute, 9609 Medical Center Drive, Room 4E442, Rockville, MD 20852 USA

**Keywords:** Grants, Review, Implementation science, Study characteristics

## Abstract

**Background:**

The National Cancer Institute (NCI) has supported implementation science for over a decade. We explore the application of implementation science across the cancer control continuum, including prevention, screening, treatment, and survivorship.

**Methods:**

We reviewed funding trends of implementation science grants funded by the NCI between 2000 and 2012. We assessed study characteristics including cancer topic, position on the T2–T4 translational continuum, intended use of frameworks, study design, settings, methods, and replication and cost considerations.

**Results:**

We identified 67 NCI grant awards having an implementation science focus. R01 was the most common mechanism, and the total number of all awards increased from four in 2003 to 15 in 2012. Prevention grants were most frequent (49.3%) and cancer treatment least common (4.5%). Diffusion of Innovations and Reach, Effectiveness, Adoption, Implementation, Maintenance (RE-AIM) were the most widely reported frameworks, but it is unclear how implementation science models informed planned study measures. Most grants (69%) included mixed methods, and half reported replication and cost considerations (49.3%).

**Conclusions:**

Implementation science in cancer research is active and diverse but could be enhanced by greater focus on measures development, assessment of how conceptual frameworks and their constructs lead to improved dissemination and implementation outcomes, and harmonization of measures that are valid, reliable, and practical across multiple settings.

## Background

The National Cancer Institute (NCI) has long supported innovative cancer prevention and control research that has produced numerous efficacious interventions for improving cancer screening, tobacco cessation, and promoting nutrition, physical activity, and sun safety [1]. However, traditional generation and evaluation of evidence in cancer control is often slow, costly, or insufficiently generalizable to provide decision-makers with useful, effective strategies for cancer control in clinical, community, and policy settings [2]. Inadequate attention to the implementation process has resulted in an enormous gap between evidence generated and actions taken to implement such evidence into routine clinical and public health practice [3]. Therefore, the usefulness of efficacious interventions developed under highly controlled research conditions has been undermined by the difficulties to translate them into complex, real-world settings. Implementation science is vital to reach the ultimate goal of improving research impact to improve cancer outcomes in the broader population, thereby achieving a return on scientific investment. While there are several definitions for implementation science, Eccles and Mittman define implementation science as “the scientific study of methods to promote the systematic uptake of research findings and other evidence-based practices into routine practice, and, hence, to improve the quality and effectiveness of health services and care [4].”

To bridge the discovery-to-delivery gap, the National Institutes of Health (NIH) has made translational science a research priority. In 2006, NIH launched the Clinical and Translational Science Awards (CTSA) and other funding programs to address the development and implementation of national standards, best practices, and infrastructure support for translational research [5-7]. Within the CTSA announcement, translational science defines two areas of translational research: 1) “the process of applying discoveries generated during research in the laboratory, and in preclinical studies, to the development of trials and studies in humans”, and 2) “research aimed at enhancing the adoption of best practices in the community [8].” Despite both areas falling under the same translational research heading, the two stages of research require different methods, measures, and outcomes; the first utilizes methods to move from fundamental biology to clinical application, while the second requires methods to map the complexity of clinical and community settings to identify the optimal fit of research within these real-world contexts. Studies of dissemination (i.e., actively spreading evidence-based interventions (EBIs) to the target audience via determined channels using planned strategies) and implementation (i.e., putting to use or integrating evidence-based interventions within a setting) fall within this second translational research area, often referred to as implementation science [9]. Implementation science emerged as a response to these distinct scientific needs to formulate strategies for promoting adoption of EBIs into clinical and public health practice, effectively reaching targeted populations, and understanding implementation processes across settings. Studying such strategies can yield important research findings that can be applied in routine care to improve cancer outcomes.

The NCI has been active in developing and promoting research grant opportunities as well as training institutes and workshops and providing institutional support for implementation science. The NCI’s support for this research field, also referred to as dissemination and implementation (D&I) research, began through administrative supplements funded from 2001 to 2008. Initially, supplements supported dissemination and implementation research of behavioral interventions. In 2006, the NCI expanded the scope of D&I research supplements to include D&I of surveillance research. During this 7-year period, the NCI successfully awarded 20 1-year, non-competitive administrative supplements of approximately $100,000 to $220,000, total costs, to investigators with active parent research project grants (R01s), research program project grants (P01s), and research project cooperative agreements (U01s) [10]. The supplement program’s success led to the NCI’s co-sponsoring the initial Trans-NIH Program Announcements on D&I Research in Health (DIRH PAR) in 2006 with eight other institutes and centers. Currently, 16 institutes and centers participate in the trans-NIH funding announcement and they have awarded 116 research grants between 2006 and 2013. Additionally, NIH has supported seven annual conferences focused on D&I and ten annual week-long trainings on D&I Research in Health [11,12]. Funding opportunities and trainings have contributed to the tremendous growth of implementation science literature in peer-reviewed journals such as *Implementation Science* and *Translational Behavioral Medicine*. While interest in implementation science has grown, the field is still an emerging and evolving discipline.

Through this period of growth, the field of implementation science has largely moved from a set of studies chronicling the many barriers and facilitators to successful adoption, uptake, and sustainability, to one focused on designing strategies to improve the transportation of EBPs into a myriad of settings. The past decade has seen movement toward the comparison of implementation strategies [13] to “implementation as usual” and more recently to the comparison of multiple active strategies. Simultaneously, researchers have been advancing the methods and measures of implementation science [14], working to increase the rigor and robustness of these complex processes.

Given the rapid growth and interest in the field of implementation science across the NIH, a review of the NCI extramural grant portfolio is timely. The purpose of this paper is to a) describe the funding characteristics and trends in implementation science supported by the NCI between 2000 and 2012, b) summarize study characteristics of implementation science funded by the NCI during that period, and c) discuss current implementation science needs and future opportunities to improve cancer prevention and control outcomes. This review identifies scientific gaps in the NCI extramural grant portfolio and encourages research needed to advance the field of implementation science.

## Methods

We searched for grants awarded between fiscal years 2000 and 2012 using an internal NCI extramural database, Portfolio Management Application (PMA), to identify NCI-funded implementation science grants. Given there are no codes to identify implementation science within PMA, we used a text search to identify NCI grants in this area. To guide selection of search terms, we adopted the NIH definition of dissemination as “the targeted distribution of information and intervention materials to a specific public health or clinical practice audience” and implementation as “the use of strategies to adopt and integrate evidence-based health interventions and change practice patterns within specific settings” [6]. Our search query was overly inclusive to capture all potentially relevant research projects related to implementation science. This query included abstracts and titles of all awarded NCI grants and competing supplements using multiple combinations of the following terms: *dissemination*, *implementation*, *diffusion*, *pragmatic research*, *translation research*, *and translational research*. Any duplicative grants were removed. An NCI staff member compiled and reviewed project abstracts from the initial search results to determine potential relevance to implementation science. A second NCI staff member reviewed a random 10% sample of all abstracts for quality control. Because information on individual non-competing administrative supplement awards is scarcely available within the NCI extramural databases, these awards were not included in our review. Additional analysis of a subset of non-competing implementation science administrative supplements are available elsewhere [15].

Eight NCI Implementation Science team members piloted a coding process on three implementation science grant applications not included in this analysis to establish reviewer consensus on coding criteria. Full applications for all grant awards identified as potentially relevant were reviewed by NCI team members with expertise in IS and extramural grants. The eight members participated in the content review and were randomly paired to assess applications. Once paired, reviewers independently assessed the full grant application including specific aims, methodology, and intended outcomes to determine inclusion or exclusion from the analysis. To be included in our analysis, the awards had to focus on topics within the cancer control continuum [16], have either a dissemination or implementation research objective, and involve human subjects. Applications were excluded if they were administratively focused (e.g., solely capacity building) and/or did not meet at least one of the following criteria: a) involve a human component, b) focus on cancer control continuum topics, or c) have either a dissemination or implementation research objective. All exclusions were based on the consensus of the paired reviewers. If there was no consensus, a third person (M.A.S.) reviewed the grant and final exclusion decisions were negotiated.

Included research projects were coded by paired reviewers working independently on the same data. Clear guidelines and codebook were developed by the authors so codes could be applied consistently to several categories including cancer prevention and control topic areas (e.g., breast cancer screening, diet/nutrition, informed decision-making, public health genomics, treatment), stated implementation science objectives (e.g., dissemination: an active approach of spreading EBIs to the target audience via determined channels using planned strategies; adoption: the decision of an organization or a community to commit to and initiate an EBI; implementation: the process of putting to use or integrating EBIs within a setting; sustainability: extent to which an EBI can deliver intended benefits over an extended period of time after external support from the donor agency is terminated) [9], translation research phase T2–T4 (T2 = efficacy studies, T3 = effectiveness studies, T4 = public health outcome studies) [6], study design (e.g., randomized control trial, group randomized trial, simulation), setting (e.g., community, faith based, home based), sustainability considerations (e.g., capacity building, cost analysis), replication indicators (e.g., monetary and/or non-monetary costs), use of implementation science frameworks or models (e.g., diffusion of innovation, Reach, Effectiveness, Adoption, Implementation, Maintenance (RE-AIM), Chronic Care Model), and analytic methods (e.g., qualitative, quantitative, cost-effectiveness). Multiple coding assignments within classification categories were allowed within each award. For example, an award could be coded for both qualitative and quantitative analytic methods. For center grants (P50s), only the implementation science component of the grant was classified by the aforementioned categories. All coding information for each grant award was collected via SurveyMonkey and extracted into Stata 12.0 for data cleaning and analysis. We provide descriptive statistics on both study and funding characteristics and describe trends over time.

## Results

**Figure 1:**
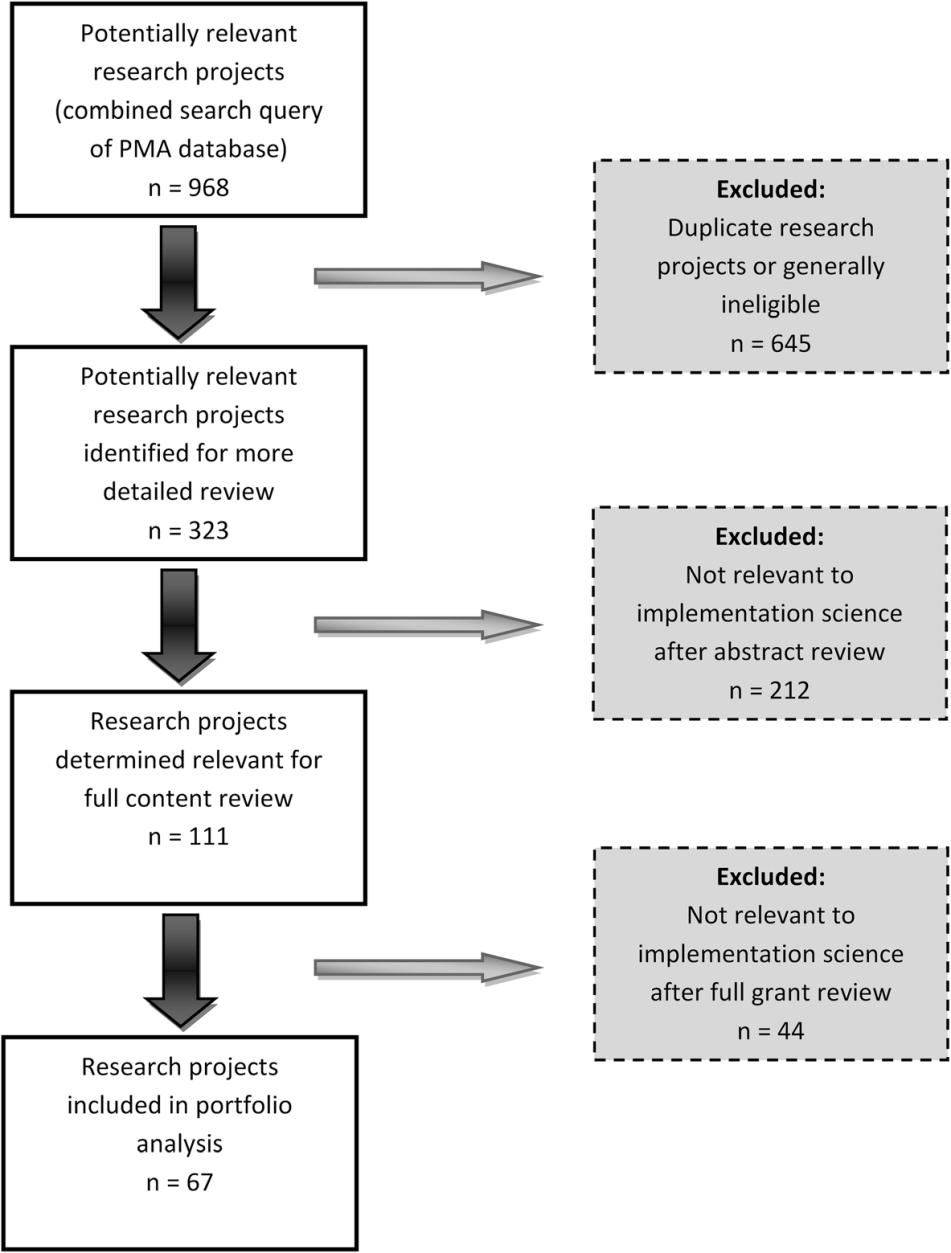
**Schematic summary of the selection process used in the portfolio analysis grant review.**

A total of 968 grants were retrieved from our combined query search. We removed all duplicative or ineligible grant awards, leaving 323 potentially relevant awards. Of the 323 awards, 111 were determined relevant to implementation science. Primary reasons for exclusion included 1) no stated dissemination or implementation research objective or 2) the implementation science component was administratively focused (i.e., infrastructure development or capacity building to conduct research). After full review, 67 grant awards were deemed implementation science relevant for our analysis (Figure 1).

The majority (70%) of included awards were large research project grants (R01s—up to 5 years of funding for discrete research project). Fifteen were small or developmental research projects (R03s, R21s—up to 2 years of funding for developmental, feasibility or pilot study), three were center grants (P50s—for large, multi-project specialized centers), one was a cooperative agreement (U01—similar to R01 but with substantial programmatic involvement from the funding agency), and one was a small business innovation research grant (R43 to stimulate technological innovation in the private sector). No competitive supplements were found to be implementation science relevant for our analysis largely because these were focused on dissemination or implementation activities and not research per se. Fewer than half (43%) of the implementation science grant awards funded between 2000 and 2012 were in response to the D&I Research in Health Program Announcements (DIRH PAR) [7,17,18] (Table 1). Of these, the majority (69%) were R01 research projects. The remaining grants funded through the DIRH PAR were small or developmental research grants, including R21s (28%) and R03s (3%). Similarly, of those grants awarded outside of the DIRH PAR, the majority (71%) were R01 research projects, which included nine investigator-initiated grants (awarded before 2009), eight grants submitted under the parent R01 (from 2009 to 2012), and 10 grants in response to specific program announcements. The DIRH PAR success rate for new and competing grants between FY 2000 and FY 2012 was 24.3% whereas the success rate for new and competing grants during the same time period for NCI cancer prevention grants (funded by the NCI’s Division of Cancer Control and Population Sciences) as a whole was 20.2% (including only R01s, R21s, and R03s, the only three mechanisms granted under the DIRH PAR.) While this is a small difference, looking only at R01s, the difference is greater: 26.7% (for DIRH) to 19.1% (NCI as a whole). Success rates for R03s and R21s are comparable between the two groups (15% for R21s, ~30% for R03s).

**Table 1:** **NCI**-**funded implementation science awards by program announcement and grant mechanism**, **fiscal years 2000**-**2012**

**Announcement**	**Mechanism**	**Funds**	**Number of awards (%)**	**Average cost per grant**	**Average cost per grant per year**
DIRH PAR					
	R01	$25,157,077	20 (69)	$1,257,854	$545,627
	R21	$2,778,562	8 (28)	$347,320	$187,859
	R03	$150,000	1 (3)	$150,000	$75,000
	Totals	$28,085,639	29		
Other announcements					
	R01	$63,790,377	27 (82)	$2,362,607	$564,723
	R21	$952,411	4 (12)	$238,103	$119,051
	R03	$300,500	2 (6)	$150,250	$75,125
	Totals	$65,043,288	33		
Overall					
	R01	$88,947,454	47 (76)	$1,892,499	$556,597
	R21	$3,730,973	12 (19)	$310,914	$164,923
	R03	$450,500	3 (5)	$150,167	$75,083
	Totals	$93,128,927	62		

Funding for projects recently awarded in 2013 are not included.

*DIRH PAR* Dissemination and Implementation Research in Health Program Announcement.

Only three awardees were new investigators, all of whom were funded through the DIRH PAR. The proportion of new investigator awards during the period FY 2000–2012 at NCI as a whole was 24.2% whereas for the DIRH PAR alone, it was 43.8%. This includes all awarded, pending and to be paid new and competing R01 grants. If restricting to awarded grants only, the proportions are 20% and 25%, respectively. Over a quarter (28%) of the 67 total NCI-funded grants came from four institutions: University of Washington St. Louis (*n* = 7), Dana Farber Cancer Institute (*n* = 4), Kaiser Foundation Research Institute (*n* = 4), and University of North Carolina Chapel Hill (*n* = 4), three of which were Cancer Prevention and Control Research Network (CPCRN) grantees. The CPCRN is co-funded by NCI and CDC and focuses on increasing D&I research collaborations.

**Figure 2:**
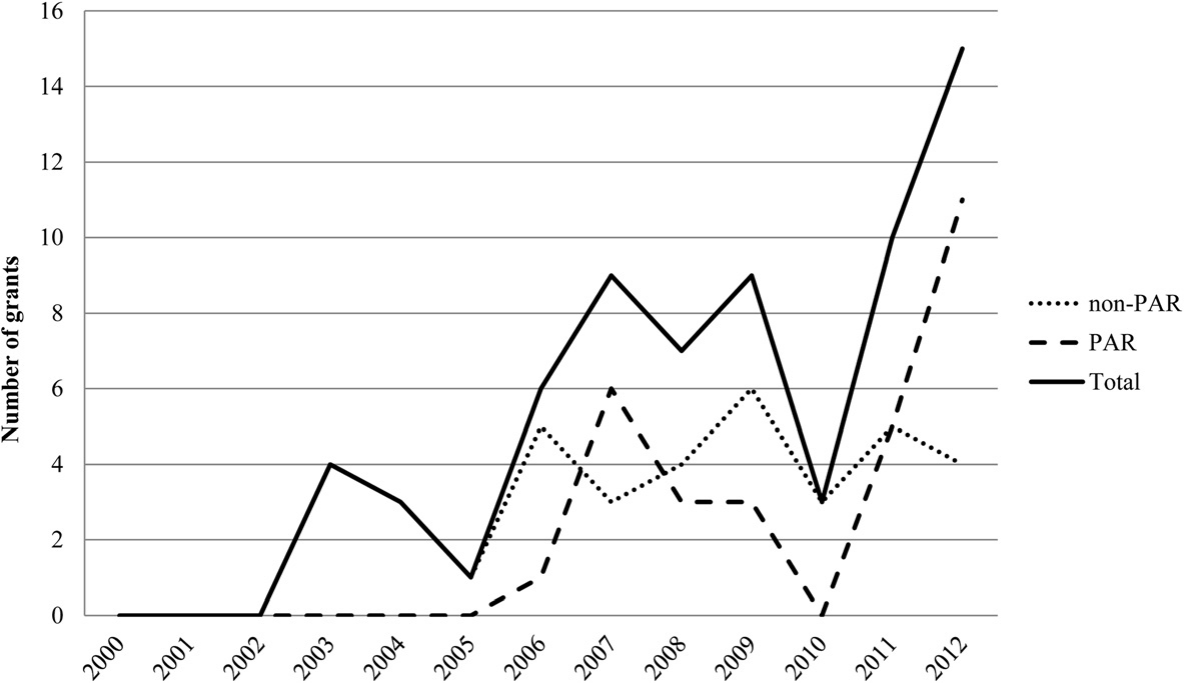
**Trends in NCI-** **funded grants in implementation science by funding announcements,** **fiscal years 2000–** **2012.** Trends in grants awarded in implementation science funded by the National Cancer Institute (NCI), fiscal years 2000–2012, overall and for the Dissemination and Implementation Research in Health Program Announcement versus all other NCI funding announcements.

**Figure 3:**
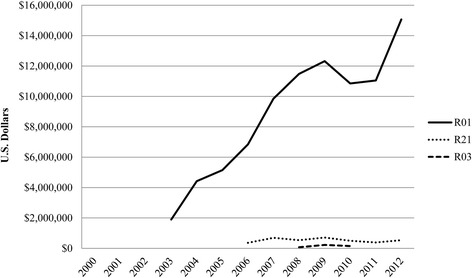
**Funding trends of NCI-** **funded implementation science by grant mechanism,** **fiscal years 2000–** **2012.** Funding trends of implementation science by grant mechanism (R01, R21, R03) funded by the National Cancer Institute, fiscal years 2000–2012.

The NCI awarded approximately $89 million in total costs toward implementation science through the R01 mechanism, averaging $557,000 per grant per year. During the same time period, exploratory research grants (R21) funded $3.7 million in total costs, averaging $165,000 per grant per year, while the small grant program (R03) received $450,500 in total costs, averaging $75,000 per grant per year (Table 1). The number of NCI-funded implementation science grants has nearly quadrupled in the last decade from four in 2003 to 15 in 2012 (Figure 2), with peaks in 2006, 2009, and 2011/2012. However, the DIRH PAR was not always the primary funding announcement supporting IS over this time period. NCI funding for implementation science consistently increased for R01 research projects from $1.9 million in 2003 to $15.1 million in 2012, while R03 and R21 awards remained relatively flat between 2006 and 2012 (Figure 3). To our knowledge, no implementation science relevant grants were funded by NCI between 2000 and 2002.

A wide range of implementation science has been funded by the NCI, spanning the cancer control continuum and addressing a variety of cancer sites and relevant risk factors (Table 2). The majority of grant awards addressed cancer prevention (49%) or screening (40%), with fewer studies examining cancer treatment (4%) and survivorship (6%). Cross-cutting applications addressed cancer surveillance, information services, and EBIs broadly defined. The most common topic area of awarded grants was tobacco control (39%), followed by colorectal cancer (30%), breast cancer (18%), and cervical cancer screening (12%). Regarding core implementation science objectives, just under half of the grants (46%) addressed dissemination research objectives and less than a third (30%), adoption. Most awards (79%) included an implementation component. Although sustainability was a stated objective in 26 awards (39%), 39 awards (58%) included at least one sustainability consideration, such as capacity building (30%), intervention maintenance (37%), or cost analysis (22%).

**Table 2:** **Number and percentage of NCI**-**funded implementation science research grants by study characteristics**, **fiscal years 2000**-**2012**

**Characteristic**	**Number of grants** ^**a**^	**Percent of grants** ^**a**^
**Cancer control continuum**		
Prevention	33	49.3
Screening	27	40.3
Diagnosis	0	0.0
Treatment	3	4.5
Survivorship	4	6.0
Cross-cutting	7	10.4
**Types of cancer/risk factors**		
Colorectal cancer	20	29.9
Breast cancer	12	17.9
Cervical cancer	8	11.9
Tobacco	26	38.8
Physical activity	6	9.0
Diet/nutrition	5	7.5
Sun safety	5	7.5
Public health genomics	4	6.0
Obesity	3	4.5
**Implementation science objectives**		
Dissemination	31	46.3
Adoption	20	29.9
Implementation	53	79.1
Sustainability	26	38.8
**Sustainability indicators**		
Capacity building	20	29.9
Maintenance	25	37.3
Cost analysis	15	22.4
**Place on T2–T4 continuum**		
T2 (efficacy studies)	8	11.9
T3 (comparative effectiveness)	61	91.0
T4 (outcomes research)	8	11.9
**Study settings**		
Community	18	26.9
Faith based	3	4.5
School based	6	9.0
Clinical	35	52.2
Workplace	10	14.9
Home based	3	4.5
Online	7	10.4
**Collaborative processes**		
Yes	60	90
No	7	10
**Study design**		
Experimental, randomized control trial	39	58.2
Experimental, nonrandomized trial	5	7.5
Observational	21	31.3
Modeling	2	3.0
**Methods used**		
Qualitative	54	80.6
Quantitative	59	88.1
Comparative effectiveness	35	52.2
Cost analysis	18	26.9
Simulation models	2	3.0
Network analysis	1	1.5
**Frameworks/models**		
RE-AIM	23	34.3
Diffusion of Innovation	26	38.8
Organizational Change	7	10.4
Systems/Network Theories	5	7.5
Chronic Care Models	7	10.4
Model of Diffusion in Service Organizations	3	4.5
**How the model was used**		
Intervention design	48	71.6
Formative research	22	32.8
Measured variables	41	61.2
Only stated	5	7.5
**Replication costs considerations**		
Yes	33	49.3
No	31	46.3
NA	3	4.5

^a^Numbers add up to more than 67 (100%) in some cases because a given grant may fit into more than one category.

The vast majority (91%) of awards were classified as T3 studies. Only eight awards (12%) were classified as T4 studies, half of which were cross-classified as T3. Eight awards (12%) were classified as T2 studies, six of which were cross-classified as T3. The most common study setting was clinical (52%), followed by community (27%), workplace (15%), online (10%), school based (9%), and faith or home based (4.5% each). Collaborative processes (i.e., including stakeholder engagement, community-based participatory research, patient engagement, designing for dissemination, and team science) were used in the vast majority of awards (90%). The most common study design proposed was the experimental, randomized controlled trial (58%). Nearly one third (31%) of grant awards were observational, and only a handful were experimental, nonrandomized trials (7%) or modeling studies (3%). Units of analyses included both individual level (73%) and group level (63%). The majority of grant awards planned to use qualitative (81%) or quantitative (88%) methods, many of which used both (69%). The most frequently named conceptual frameworks were Diffusion of Innovations (39%) and RE-AIM (34%). The majority of awards used frameworks for intervention design (71.6%) and measured variables (62.1%). Almost half of the awarded grants (48%) incorporated more than one framework or used only one framework (46%). Five awards did not use a framework. All study characteristics are described in Table 2.

## Discussion

The purpose of this paper is to describe the funding characteristics and trends of implementation science supported by the NCI between 2000 and 2012, summarize characteristics of implementation science funded by the NCI, and discuss current implementation science needs and future opportunities that may improve cancer prevention and control outcomes. Our review assessed 67 implementation science grant awards funded by the NCI between fiscal years 2000 and 2012 across a variety of cancer control topics, study designs and methods, and translational phases of research demonstrating a diverse set of research projects, reflecting a rapidly evolving area of science. Our portfolio analysis revealed several interesting findings. First, the number of NCI-awarded grants supporting implementation science has increased tremendously over a 10-year period in both number of grants and total funding amount, largely through the R01 mechanism. This increase indicates the growing interest in implementation science cancer control research and the ability of investigators to successfully compete for implementation science grant awards. We also noticed continued success and growth in the number of awards after the introduction of the DIRH PAR in 2006 and again in 2009, when a standing study section in D&I research replaced a special emphasis panel. Moreover, the initiation of the annual Training Institute for Dissemination and Implementation Research in Health in 2011 likely contributed to further growth in the number of awards in 2011 and 2012, as it made available to the field a large collection of implementation science lectures and tools. However, the number of new investigators supported by NCI grant awards in implementation science was dismally low during this time period. These findings suggest limited training opportunities available in academic programs for implementation science topics. Recent NIH efforts are addressing this challenge by investing in training the next generation of implementation science investigators [19]. Although current NIH-sponsored trainings do not meet the demand and growing interest in this field, additional training strategies, including webinars, graduate courses, and continuing education programs, may support new investigators [20]. We also found that small or developmental research projects (i.e., R03s and R21s) were few and remained relatively flat in funding over time. This does not necessarily mean that the NCI is not supporting R03s and R21s in implementation science. Rather, the NCI could be receiving few applications using these mechanisms for implementation science. This is consistent with what other NIH institutes and centers have reported in terms of funding small developmental grants [21]; thus, the NCI is not unique in this respect. Moreover, comparing the success rates of R03s and R21s through the DIRH PAR specifically compared with cancer prevention and control grants in general, we found that the rates are comparable: success rates for R03s were 33.3% for DIRH, 30.2% in general; and for R21s, 15.0% for DIRH, 15.3% in general. Future applicants could use these mechanisms to focus on developmental and observational studies, implementation feasibility research, and development of measures that are valid, reliable, and practical given the limited budget provided under these mechanisms [22-24]. In addition, as a range of data sources have become available for mining by researchers, retrospective analyses of implementation efforts and simulation studies of potential approaches to implement effective practices can be fostered [17,18].

Our review illustrates the breadth of implementation science cancer control research spanning across 14 topic areas, with tobacco control as the most common topic. This finding may be reflective of the advanced state of the science in policy and population-level intervention research in tobacco control [25], making it more amenable to T3–T4 translational research. In contrast, fewer studies focused on obesity, which is a more recent public health problem compared with tobacco control, and thus, fewer policies and evidence-based interventions may exist. Similarly, studies on cancer prevention and screening were more common than those of survivorship, perhaps because of the numerous existing interventions in cancer screening compared with those in cancer survivorship. Based on the NCI-sponsored database of Research-Tested Intervention Programs (RTIPS) (http://rtips.cancer.gov/rtips/index.do), there are more than twice as many evidence-based interventions in tobacco control compared with obesity prevention and three times as many in cancer screening compared with cancer survivorship.

We found diversity across study designs, methods, and conceptual frameworks used. The majority of awards proposed a randomized control trial design, with the unit of analyses at both group and individual levels. It would be interesting to assess how such trials balance rigor with practical design features (i.e., pragmatic trial criteria), both within the award and in publication of study results [26]. This balance reflects intervention feasibility and, if feasible, increases the likelihood that they will be adopted by similar settings. Pragmatic trials and accompanying tools leverage rigor and relevance to study effectiveness of interventions in real-world settings. Such trials are commonly used in implementation science to determine intervention effects, for whom, and under what conditions [27,28].

We examined the composition of D&I research objectives (i.e., dissemination, adoption, implementation, sustainability) in our portfolio analysis. These objectives are distinct concepts that make up parts of the continuum of implementation science. All of these concepts are important for translating evidence into practice, so it is important to understand which of these objectives are being studied in the context of implementation science and which may be lacking or under-evaluated. While most studies examined implementation and roughly half, dissemination, less than a third examined adoption. Adoption is distinct from implementation in that it assesses the degree to which relevant organizations or settings decide to adopt an intervention or program while implementation assesses the strategies used to implement the intervention or program within organizations or settings. Although only 38% examined sustainability explicitly, we were encouraged to find that about half of grant awards proposed cost indicators and/or included replication considerations.

With this improved coverage across multiple stages of implementation science, we see opportunities for cancer control implementation science researchers to advance key areas of under-representation in the field. Few studies have examined the alternate to sustainability, that is, what factors and strategies might influence rational attempts to discontinue or de-implement cancer control interventions. In addition, implementation science studies have rarely attempted to implement multiple EBPs across the cancer control continuum into a “braided” system of care. Finally, we saw limited capture of the way in which interventions adapt and evolve over time; a dynamic view of adoption, implementation, and sustainability in future studies may present a more naturalistic perspective on the fit of interventions within community and clinical contexts [29]. These and other priority questions highlighted in the current PARs [7,17,18] remain as horizons for the field and opportunities for subsequent investigation.

Almost all awards proposed both qualitative and quantitative methods. These are promising findings given that the use of mixed methods is critical to adapt EBIs, understand the context in which interventions are implemented, and identify factors associated with successes and failures of implementation efforts [30,31]. Unsurprisingly, Diffusion of Innovations and RE-AIM—two of the more common frameworks within the implementation science field—were the most widely reported models in our sample. However, most grant awards reported using more than one implementation science conceptual framework, and the primary use of frameworks was for intervention design, while 39% of studies did not use IS frameworks in dissemination or implementation measurement. This may reflect the problem that very few frameworks offer construct measures or empirical data supporting key variables necessary for effective dissemination and implementation research, despite the large number of implementation science conceptual frameworks (over 61) [32]. Reliable, valid, and pragmatic measures for theoretical constructs are urgently needed to standardize measurement and advance implementation science systematically [20,23,24]. Several national initiatives, including the NCI Grid-Enabled Measures Portal [23,33], North Carolina Translational and Clinical Sciences Institute [34], and Seattle Implementation Research Collaborative [35], are pooling available measures and seeking consensus from the research community to harmonize implementation science measurement.

Some study limitations warrant mention. Possibly, as with any portfolio review, not all relevant grants were identified and included in this review. To minimize the likelihood that relevant grants were omitted, we used multiple and broad search terms, multiple coders, and clearly defined inclusion and exclusion criteria. We did not include non-competitive supplemental awards in the analysis as they are not available as independent items within the PMA database. Additionally, the process for ascertaining inclusion criteria and coding of grants required interpretation and judgment. We addressed these issues by inter-rater consensus of any disagreements, both for exclusion of full grant review and coding inconsistencies, but we did not measure nor report on coding reliability. We also did not examine publications resulting from grant awards reviewed for this analysis. Therefore, it is uncertain how much of the proposed research in the awards was actually completed and whether changes were made to study design post-funding. While this is a comprehensive review, it is not an exhaustive review of all cancer implementation science. Other foundations and agencies fund similar projects. Also, we did not evaluate the impact of implementation science awards. Despite these limitations, we believe this analysis provides a unique overall snapshot of implementation science funded by the NCI.

## Conclusion

Addressing the discovery-to-delivery gap and strategically investing limited national resources in translatable health science has never been more critical. Our portfolio analysis demonstrates growing interest in implementation science in the context of cancer control and prevention research. While we are encouraged by the number of awarded grants across diverse topics, more focused and systematic implementation science is needed to address the field’s complex, multilevel, and interdisciplinary nature. This includes systematic reviews of available implementation science measures, development of valid, reliable, and practical measures, and empirical assessment of model constructs relative to health outcomes. Additionally, our portfolio analysis found very few grants focused on exploratory research to address these gaps and few new investigators were awarded implementation science grants. Much work remains to systematically improve methods to contribute significantly to cancer control research at the NCI. We conclude that NCI-funded implementation science in cancer control research is active and diverse but could be enhanced by greater focus on measure development, assessment of how conceptual frameworks and their constructs lead to improved dissemination and implementation outcomes, and harmonized implementation science measures that are valid, reliable, and practical across multiple settings.

## Abbreviations

CTSA: Clinical and Translational Science Award
D&I: Dissemination and Implementation
DIRH PAR: Dissemination and Implementation Research in Health Program Announcement
EBI: Evidence-based intervention
NCI: National Cancer Institute
NIH: National Institutes of Health
PAR: Program Announcement (with Review)
PMA: Portfolio Management Application
